# Right ventricular ejection fraction, measured during inter-stage cardiac magnetic resonance imaging, predicts outcome for patients with hypoplastic left heart syndrome

**DOI:** 10.1186/1532-429X-11-S1-O48

**Published:** 2009-01-28

**Authors:** Marina L Hughes, Katherine Brown, Vivek Muthurangu, Victor Tsang, Andrew Taylor

**Affiliations:** 1 Great Ormond Street Hospital for Children, London, UK; 2 Institute of Child Health and Great Ormond Street Hospital for Children, London, UK

**Keywords:** Hypoplastic Left Heart Syndrome, Norwood Procedure, Short Axis Cine, Phase Contrast Flow, Short Axis Cine Image

## Background

Since 2003 our unit has adopted an imaging protocol for all infants with hypoplastic left heart syndrome (HLHS), which includes CMR imaging for inter-stage assessment prior to the formation of a bidirectional cavo-pulmonary shunt. The aim of this study was to assess whether the CMR data acquired during this protocolised follow-up could help to stratify the risk for these patients.

## Methods

We assessed all locally followed patients, who had undergone the Norwood procedure for HLHS between January 2003 and May 2008, and who had undergone CMR imaging according to unit protocol.

Imaging was performed under general anaesthetic, using a 1.5 T MR scanner, and a combination of cine sequences, phase contrast flow sequences and gadolinium-enhanced MR angiography. From short axis cine images, manual segmentation of the ventricles was completed, giving ventricular volumes, ejection fraction and cardiac output (CO). Arterial measurements were made from the isotropic angiographic data using 3D analysis software. The pulmonary artery (PA) measurements were made at the proximal native vessel and distally, just prior to the 1st lobar branch. Aortic measurements were made at the narrowest point of the proximal descending aorta (CoA) and at the diaphragmatic-level descending aorta. At each site the shortest and orthogonal cross-sectional diameters were averaged, to correspond with conventional 2D measurement methods. Additionally, the exact cross-sectional area of the vessel at each point was measured using manual planimetry. The coarctation (CoA) index was defined as the (CoA measurement/diaphragmatic aorta measurement), for vessel diameter and planimetered area respectively.

The primary outcome measure was survival to analysis date (1^st^ October 2008). Secondary, functional outcome measures were RV ejection fraction (RVEF), and CO.

## Results

**Figure 1:**
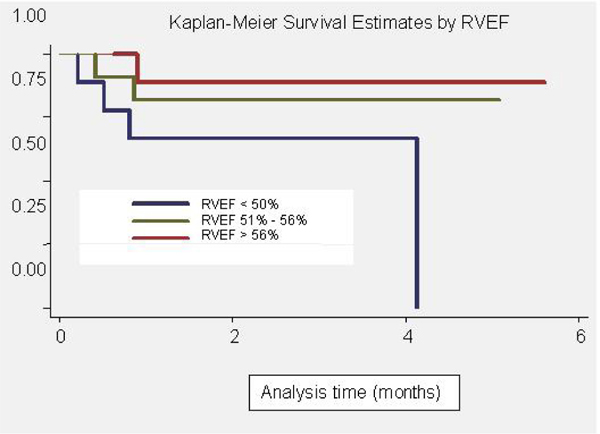
**Kaplan-Meier survival estimates by RVEF**.

A total of 30 patients comprised the cohort of survivors of the first stage Norwood procedure, undergoing protocolised CMR. Of these 30 patients, 15 had a Sano-type, and 15 a conventional Norwood. The median age and weight at CMR scan was 91 (33 – 291) days, and 5 (3.2 – 11) kg respectively. The planimetered area index of vessels was smaller than averaged orthogonal diameter index. The mean ratio of the CoA diameter index to the CoA area index was 1.4 (95% CI 1.25 – 1.55), indicating that diameter measurements may underestimate true narrowing. The PA size and indices of proximal stenosis did not correlate with outcome. The median CoA area index for all patients was 0.52 (0.22 – 1.0). Twenty-one (70%) patients had a CoA area index < 0.7, with no difference between the Sano-type and conventional Norwood surgery. There was a significant correlation between the CoA diameter index and cardiac output -1.09 (95%CI -2.17 – -0.02) (p = 0.04), but the CoA size indices were not correlated with RVEF. The median RVEF for this cohort was 53% (30 – 81%). Eight patients had RVEF < 50%. There were 7 deaths in this cohort, during a total follow-up time of 67 person years. The RVEF was strongly predictive of death, with a hazard ratio 0.92 (95% CI 0.86 – 0.99) (p = 0.02). Figure 1 demonstrates a Kaplan Meier survival curve with patients stratified by RVEF. Other factors, such as the age at time of MRI, the type of Norwood, the CO, the CoA and the PA indices did not predict death.

## Conclusion

This study shows that death is a more likely outcome in HLHS patients with a lower RV ejection fraction at inter-stage CMR. Other CMR-measured factors such as CoA and PA size indices did not predict outcome. Measures to preserve RV systolic function, and CMR assessment of this, should be paramount in the complex management of these patients.

